# Sweet Milk a Remedy for Dropsy

**Published:** 1858-04

**Authors:** 


ARTICLE VII.

TRANSLATED BY DR. BYFORD.

### SWEET MILK A REMEDY FOR DROPSY.

In a Warsaw medical journal, a treatise with the above caption has made its appearance, which we think should have a more general circulation.

If it should be said that our Materia Medica is already rich in remedies for this disease, we should at least welcome a remedy so simple and that may be used in almost any case, even when other remedies justly prized are not applicable, or may have failed. According to the author of this treatise, (Dr. Milosz), as he states in his introductory remarks, dropsy is for the most part but a symptom or result of many chronic cachexia and organic degenerations, which, when sufficiently developed, are beyond the reach of our skill by the use of ordinary remedies, but which he thinks are often curable by this simple remedy. He seems not to have been the first to use and recommend milk for this affection, but a paper written by Dr. Serres, in Cayol's *Revue Medicale*, 1856, recommending it, induced him to use it, and he now adds his own experience and observation upon its operation, and recommends it in the highest terms to the profession. Dr. Milosz has used milk exclusively in different forms and grades of dropsy, and with the exception of one case of *hydrops universalis* in an old cachectic man, with the very best results. The case was of a man, aged sixty years, with an ill-defined and obscure thoracic disease of a chronic character, who died six months after the remedy had been tried. The rest treated by him all convalesced, and, at present, after the lapse of several years, show no signs or trace of the disease. In the most cases, treatment was immediately begun with the milk, and in some it was resorted to after other means had been used in vain. In but a single case was diuretics and roborants useful. The milk was used exclusive of all other ingesta, uniformly and well borne by the patients, who nearly all were country people, and only when the quantity was increased up to six or eight quarts per day did it become disagreeable and was refused. The excretions, from the kidneys, alimentary canal and skin, were always augmented to a considerable degree. It should be added, the cases treated could with propriety be traced back to rheumatic affections, or imperfeet hematosis from deficient nourishment, without any apparent organic alterations.

We will here give some of Dr. Milosz' detailed cases, which seem to favor his statements.

No. 1. A man, sixty-eight years of age, took cold in January, 1853, while working out doors. He lost his appetite, and felt fatigued and oppressed. In a short time, œdema of the feet was succeeded by general dropsy. In the opinion of our author, his condition was quite critical, and he resolved to confine him to a half glass of sweet milk, morning and evening, and withdrew every other sort of alimentary substances. This produced increased alvine evacuations; and, as the quantity of milk was augmented, the kidneys were excited to copious secretions. The quantity was gradually enlarged until it reached sixteen pounds daily, upon which disgust made it necessary to somewhat lessen the dose. After three weeks, the dropsy had entirely disappeared, and the patient, in good health, returned to his home and employment.

No. 2 is a country woman, who fell sick in the latter part of her pregnancy as the result of cold, with pluerisy, which was followed by œdema of the feet. After her confinement, she was affected with inflamed breasts, which lasted for sometime, when general dropsy was developed, which, at the beginning, was treated with diaphoretic and antiphlogistic remedies without any good effect. She now began to take sweet milk, which was gradually increased in quantity, until she took two quarts in the twenty-four hours, with so much benefit, that, in six weeks, there was not a trace of the affection left. A decoction of juniper berries and aromatic wine of iron were given to the patient to take home, as accelerators to convalescence. Here the author remarks, that the circumstances of the patients may sometimes prevent the administration of a large quantity of milk, and that three glasses a day, constantly given, will often accomplish the desired end.

No. 3 was a poor widow, fifty years of age, affected with

*plica polonica;* after being exposed to cold, she was attacked with general dropsy. She was subjected to the milk-cure for five weeks with the best results. The kidneys and skin, and in a less degree, the alimentary canal, were all quickened in their excretory capacity.

No. 4 was a farmer, aged about sixty years, of cachectic appearance, who had, for a long time, labored under pulmonary catarrh. He was attacked with pneumonia, which was treated by bleeding, nitre and tart. ant., and apparently cured; but, during his convalescence, after a dysenteric diarrhœa, he became universally dropsical in a high degree. With little hope for a cure, the milk treatment, which had so often proven useful, was tried. After a few days, the distressing sense of suffocation, which gave him great suffering, was very much relieved, and in three weeks the treatment was withdrawn on account of the amelioration of the symptoms; and although it was twice necessary to recur to the remedy, a complete cure followed. He left the hospital, and died one year after, of the epidemic cholera.

The author assures us that he could bring many other cases in proof of the excellent effect of milk in dropsy, but thinks the above are a sufficient number of examples to justify a trial of it by others. How the milk operates he will not pretend to conjecture, but thinks there is no more obscurity in this respect than rests over the *modus operandi* of digitalis, squills or most of the approved anti-hydropic remedies. Some of his neighboring physicians had used the milk-cure without uniform effects, but he did not think their experiments in the least degree rendered questionable the usefulness of this valuable means of cure. At most, they only prove the necessity for further observation and experience to determine more precisely the indications for its administration, in order to make its effects uniform and reliable. In conclusion, the author states, that the milk should in the most of cases be administered very warm. We refrain from making any further remarks in regard to the above essay, only stating, that it is equally simple with the renowned citron cure. We may also mention that recently from other quarters we have heard of cases of dropsy cured by sweet milk. Let it be tried and reported upon.—E. K., *Med. Zeitung, Russlands.*

